# Pulsed laser deposited CoFe_2_O_4_ thin films as supercapacitor electrodes

**DOI:** 10.1039/d0ra02564j

**Published:** 2020-05-20

**Authors:** S. M. Nikam, A. Sharma, M. Rahaman, A. M. Teli, S. H. Mujawar, D. R. T. Zahn, P. S. Patil, S. C. Sahoo, G. Salvan, P. B. Patil

**Affiliations:** School of Nanoscience and Technology, Shivaji University Kolhapur Maharashtra – 416004 India; Semiconductor Physics, Chemnitz University of Technology 09107 Chemnitz Germany salvan@physik.tu-chemnitz.de; Department of Physics, Shivaji University Kolhapur Maharashtra – 416004 India; Department of Physics, Yashavantrao Chavan Institute of Science Satara Maharashtra – 415001 India; Department of Physics, Central University of Kerala Kasaragod Kerala – 671320 India; Department of Physics, The New College, Shivaji University Kolhapur Maharashtra – 416012 India prashantphy@gmail.com

## Abstract

The influence of the substrate temperature on pulsed laser deposited (PLD) CoFe_2_O_4_ thin films for supercapacitor electrodes was thoroughly investigated. X-ray diffractometry and Raman spectroscopic analyses confirmed the formation of CoFe_2_O_4_ phase for films deposited at a substrate temperature of 450 °C. Topography and surface smoothness was measured using atomic force microscopy. We observed that the films deposited at room temperature showed improved electrochemical performance and supercapacitive properties compared to those of films deposited at 450 °C. Specific capacitances of about 777.4 F g^−1^ and 258.5 F g^−1^ were obtained for electrodes deposited at RT and 450 °C, respectively, at 0.5 mA cm^−2^ current density. The CoFe_2_O_4_ films deposited at room temperature exhibited an excellent power density (3277 W kg^−1^) and energy density (17 W h kg^−1^). Using electrochemical impedance spectroscopy, the series resistance and charge transfer resistance were found to be 1.1 Ω and 1.5 Ω, respectively. The cyclic stability was increased up to 125% after 1500 cycles due to the increasing electroactive surface of CoFe_2_O_4_ along with the fast electron and ion transport at the surface.

## Abstract 2

Cobalt ferrite thin films were grown by PLD at different temperatures as an electrode material for supercapacitors. The films deposited at room temperature exhibited the best power density (3277 W kg^−1^) and energy density (17 W h kg^−1^) values.

## Graphical abstract

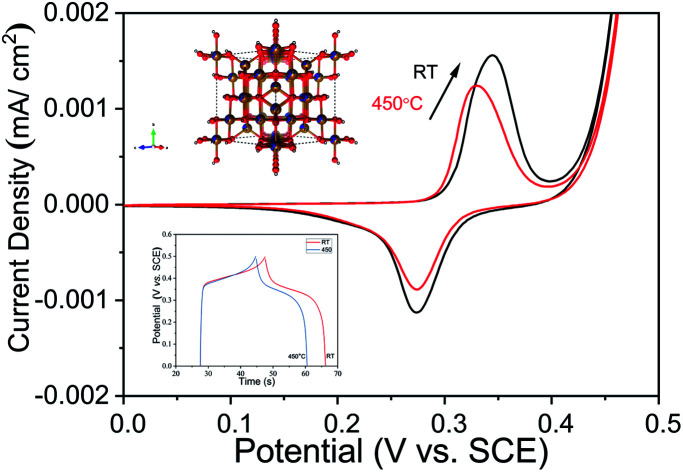

## 1. Introduction

With the endless desire for electricity and drift from the conventional power grid to renewable energy sources, the demand for efficient energy storage devices is rising exponentially. For this, the supercapacitor (SC) is an excellent solution due to its fast charging/discharging rates, high power density, long cycle life, and eco-friendly nature.^1–4^ In general, SCs can be classified into two types based on the operational charge–discharge storage mechanism: electric double-layer capacitors (EDLCs) and pseudocapacitors. EDLCs work on the principle of electrostatics, where the energy is stored by trapping ions at the electrode/electrolyte interface.^2^ A pseudocapacitor is based on the faradaic charge transfer process that utilizes reversible redox reactions and intercalation at electrodes to store charge. In particular, pseudocapacitive materials can achieve excellent specific capacitances and energy densities, and they can thus be an interesting source for constructing novel energy storage devices.^5^ However, the pseudo capacitor has a comparatively shorter cycle life than an EDLC, due to the not completely reversible redox reactions and the loss of electrical contact resulting from the disintegration of the crystal structure, which reduces electrochemical performance. This can be overcome by combining a pseudo-active material a with conductive support, also known as hybrid supercapacitors.^6^ Transition metal oxides, such as RuO_2_, Co_3_O_4_, and Fe_2_O_3_ have been considered as active materials for supercapacitors on account of their high theoretical capacitance, variable oxidation states, environmentally friendly nature, and low cost.^7,8^ Further, several researches have been focused to improve performance, storage, and energy densities for SCs by either tailoring the material properties^9,10^ and/or altering the surface^11^ of the electrode materials. Some of the prominent approaches include the electrode made of porous carbon,^12^ carbon nanotube,^13^ and metal–organic frameworks.^14–16^

**Fig. 1:**
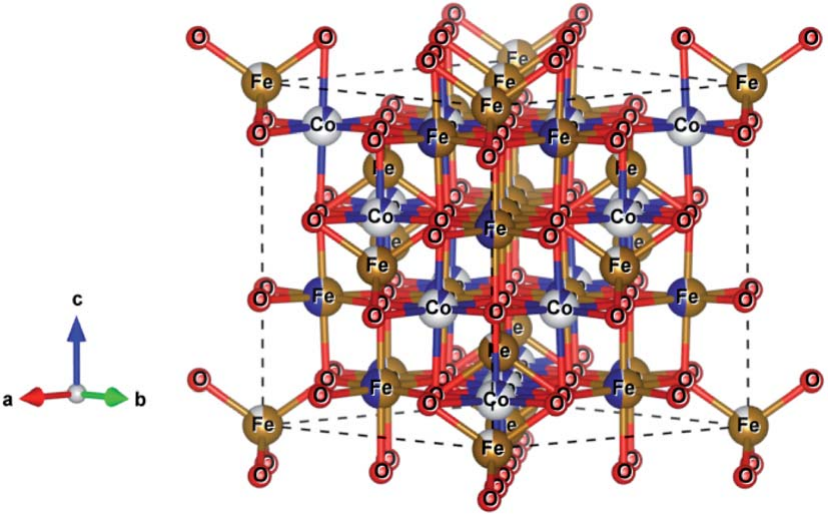
Schematic crystal structure of spinel CoFe_2_O_4_ ferrite (*Fd*3̄*m* space group) showing Co and Fe cations as grey and gold spheres and O anions as red spheres, respectively.

Recently, mixed metal oxides with spinel ferrite structure, MFe_2_O_4_ (M = Co, Mn, Zn, Mg, or Ni), were reported to have distinct hard or soft magnetic properties or even superparamagnetism, a large range of oxidation states, and chemical stability.^17^ In spinel ferrites, the divalent metal ion (M^2+^) occupies the tetrahedral site, and the trivalent metal ion (M^3+^ or Fe^3+^) occupies the octahedral position of the cubic close-packed oxygen lattice, see Fig. 1.^18,19^ D. Ravinder *et al.* reported that the ferrites have remarkably high permeability and can attain multiple redox states.^20^ These properties of ferrites were explored by several researchers who showed that the ferrite electrode has superior pseudo-capacitance.^21–23^ It is anticipated that mixed ferrites, with varying ratios of the M and Fe ions, improve the electrode performance, due to their higher number of cations for coordination sites.^24^

Among the known mixed spinel ferrites, CoFe_2_O_4_ has a variety of merits, including excellent chemical stability, efficient electrocatalytic behaviour, high specific capacitance that makes it a suitable candidate for electrodes in supercapacitors.^25–27^ To harness these advantages of CoFe_2_O_4_ for supercapacitor applications, numerous chemical methods of preparation were reported, such as solvothermal, hydrothermal, electrodeposition, and spin coating.^17,28–30^ Even though all these chemical methods are cheap, they all have issues with the reproducibility of the stochiometric composition of the deposited material. On the other hand, the potential of the pulsed laser deposition (PLD) technique, which has an excellent stochiometric reproducibility of the target material,^31,32^ has not yet been explored for the deposition of cobalt ferrite electrodes for supercapacitor application.

The present study reports the efficacy of PLD deposited CoFe_2_O_4_ thin films for supercapacitive electrode applications. The films with different crystallinity were prepared by using different substrate temperatures. Mesoporous films prepared at RT can provide a higher surface area for an electrolyte to diffuse with low transfer limitation. The low crystallinity of the electrode material has improves the exposure to active sites accessible for the electrolyte on the surface.

## 2. Experimental

Cobalt ferrite thin films were deposited on a fused quartz substrate in a PLD vacuum chamber with a base pressure of 2 × 10^−5^ mbar. A sintered cobalt ferrite target comprised of α-Fe_2_O_3_ and Co_3_O_4_ in the stoichiometric ratio was repetitively (10 Hz) irradiated with the third harmonic (*λ* = 355 nm) of an Nd:YAG laser for 6 ns pulse duration delivering a typical fluence of 2.5 J cm^−2^ on the target during deposition. The fused quartz substrate was kept at a distance of 45 mm from the target. In this way, we fabricated CoFe_2_O_4_ thin films at room temperature (RT), 350 °C, and 450 °C substrate temperature. A clear evidence of crystallisation in the X-ray diffraction studies was observed only at 450 °C substrate temperature and the electrochemical performance of the 350 °C films (not shown here) was similar to that of the RT films. Thus, for the ease of discussion, only the RT and 450 °C samples are considered for the further investigations. Thicknesses of films prepared at RT and 450 °C substrate temperature were found to be around 110 nm and 200 nm, respectively.

The crystallographic investigation of the films was performed by recording X-ray diffraction (XRD) 2*θ*–*ω* scans using a Bruker D2 PHASER X-ray diffractometer. Raman measurements were carried out using XploRA PLUS Raman spectrometer from Horiba scientific under 532 nm diode-pumped solid-state (DPSS) laser excitation using a 100×/0.9 NA objective with a laser power density of 1.33 mW μm^−2^ in a back-scattering geometry. The collected Raman signals were dispersed onto an electron multiplier charge-coupled device (EMCCD) using a 1200 l mm^−1^ grating. The sample surface morphology was examined using an Agilent 5420 atomic force microscope operated in intermittent contact mode. The electrochemical measurements were carried out in the three-electrode system using Autolab's potentiostat PGSTAT302.

For electrochemical measurements, cobalt ferrite films were used as both electrode material and current collectors. For a seamless electrical contact to the film, a small piece of silver was clipped onto the conductive cobalt ferrite film by a toothless alligator clip to connect the battery analyser. Then the assembly was dipped in the 1 M KOH aqueous electrolyte solution for electrochemical measurements. Cyclic voltammetry (CV), galvanostatic charge–discharge (GCD), and electrochemical impedance spectroscopy (EIS) were carried out with conventional three-electrode arrangement comprising platinum as a counter electrode, a saturated calomel electrode (SCE) as a reference electrode, and CoFe_2_O_4_ as a working electrode. Cyclic voltammetry measurements were carried out at various scan rates of 10–100 mV s^−1^ between the potential window of 0 V and 0.5 V. Galvanostatic charge–discharge measurements were carried out at a charging current density (0.5 mA cm^−2^) between 0 V and 0.5 V *vs.* SCE. The specific capacitance of the supercapacitor was calculated from GCD curves according to the equation^19^1
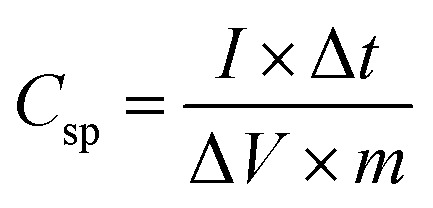
Here, *C*_sp_ is the specific capacitance (F g^−1^), *I* is the response current density (mA cm^−2^), *m* is the mass of the electrode (g), Δ*V* is the potential range (V), and Δ*t* is the discharging time.

## 3. Results and discussion

### 3.1. Structural analysis

**Fig. 2:**
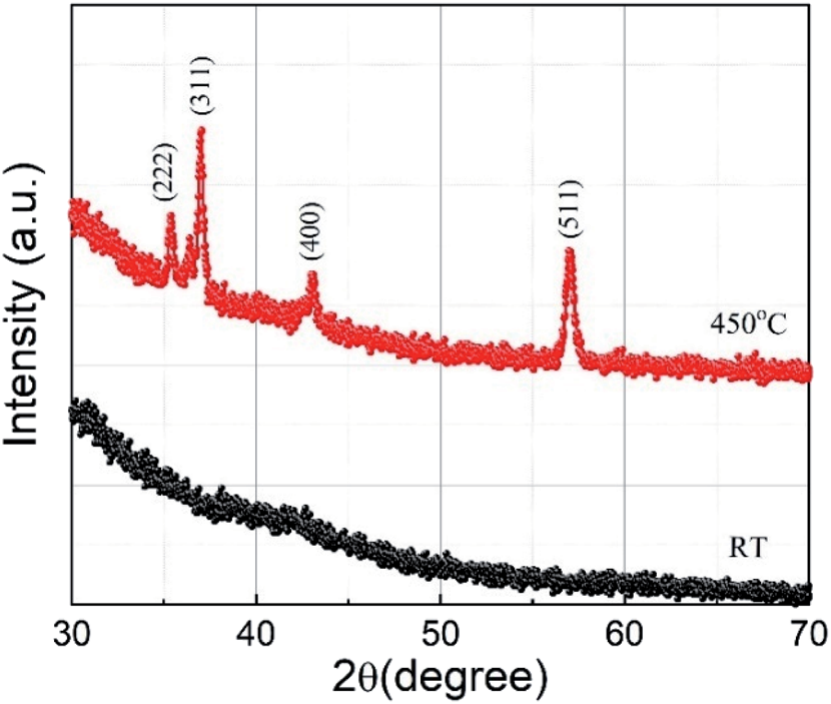
X-ray diffraction patterns recorded for CoFe_2_O_4_ thin films deposited at substrate temperatures of RT (black) and 450 °C (red). The observed peaks in the scan are labelled with corresponding crystal planes of CoFe_2_O_4_.

XRD 2*θ*–*ω* scans of the sample deposited at substrate temperatures of RT and 450 °C are shown in Fig. 2. The film deposited at RT does not show any XRD peak, implying that the film is amorphous. However, the film grown at a substrate temperature of 450 °C shows sharp diffraction peaks and the peaks observed can be indexed to the cubic Co-ferrite phase with the help of JCPDS data (ICDD, PDF 221086 for CoFe_2_O_4_) and assigned to the lattice planes (222), (311), (400), and (511) corresponding to the cubic spinel structure of CoFe_2_O_4_. The peaks with smaller intensity appearing around 36.40° and 42.52° are due to the presence of CoO (ICDD, PDF 431004 for CoO) in the sample. However, the relative intensity of the CoO peaks with respect to the CoFe_2_O_4_ peaks is minimal and the Co is thus expected not to influence the electrochemical performance of the CoFe_2_O_4_ electrode. We can also see in Fig. 2 that a lower number of peaks compared to the bulk were observed in the film. The films generally showed a preferred orientation in the (311) direction, as it is the case for many other reports of CoFe_2_O_4_ thin films.^7,33^ The lattice constant for the film deposited at 450 °C calculated using the (511) peak was found to be 8.4 Å which is comparable to the bulk crystallite value (ICDD, PDF 221086). For films deposited at 450 °C, the average crystallite size calculated by the Debye Scherrer formula using the (511) peak was found to be (19 ± 1) nm. The film deposited at RT may have grains with very small size, which could not be detected by XRD. In our previous work, reporting CoFe_2_O_4_ film preparation at different temperatures by PLD, we observed similar XRD behaviour for films prepared at RT.^34^ The selected area electron diffraction (SAED) pattern of these films prepared at RT showed diffraction rings which were identified as the Co-ferrite phase. This indicates that our films deposited at RT consist of low crystalline CoFe_2_O_4_ phase.

### 3.2. Raman spectroscopy

**Fig. 3:**
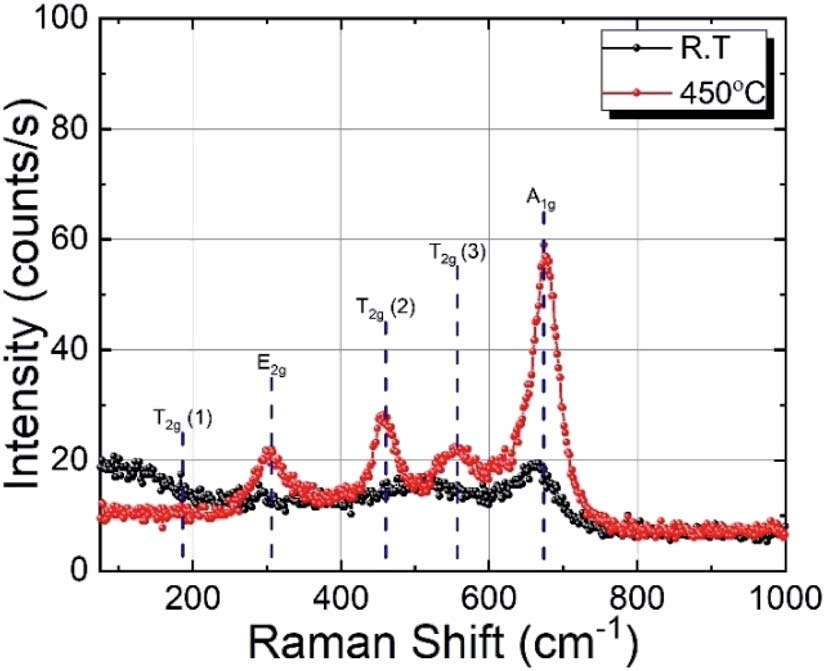
Raman spectra of CoFe_2_O_4_ thin films deposited on fused quartz substrate at RT and 450 °C.

The Raman spectra of cobalt ferrite thin films prepared at RT and 450 °C are shown in Fig. 3. Raman spectroscopy is a powerful technique in understanding crystal structures of a material down to nano-size domains. CoFe_2_O_4_ belongs to the space group *Fd*3̄*m*, predicting 39 phonon modes for this spinel structure. Among them, the five Raman active modes are A_1g_, E_g_, and 3T_2g_.^35,36^ The modes below (above) 600 cm^−1^ are attributed to the oxygen motion around the octahedral (tetrahedral) lattice sites.^37^ The broadening of the Raman modes indicating its amorphous nature is in agreement with the XRD results. The film deposited at 450 °C substrate temperature shows four prominent Raman active modes at 302 cm^−1^, 458 cm^−1^, 556 cm^−1^, and 678 cm^−1^, which correspond to E_g_, 2T_2g_, and A_1g_ phonon vibrations, respectively. The Raman active T_2g_ mode around 200 cm^−1^ is absent in the spectra, probably due to the weak Raman cross-section of this particular mode. The A_1g_ Raman modes of the film prepared at 450 °C show a slightly asymmetric shape with a shoulder at the low-frequency end. This can be explained by the cationic radii at the octahedral and tetrahedral site (either Co or Fe) and the Co/Fe–O bond distance. Since Raman spectra are sensitive to the local structural change, the distribution of the local bond length can produce this double peak-like shape in the Raman spectra.^38^ In the case of other modes, this asymmetry is not visible, which may be due to the relatively low Raman intensity. The presence of four well-resolved Raman modes in the Raman spectrum of the sample prepared at 450 °C confirms the better crystalline quality compared to films prepared at RT. The Raman results are in good agreement with the XRD results presented in the previous section.

### 3.3. Topographic analysis

**Fig. 4:**
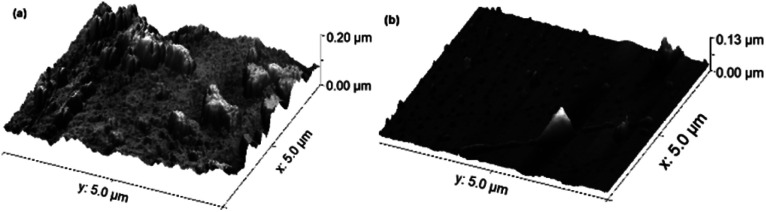
Pseudo-3D AFM images of CoFe_2_O_4_ thin films deposited at substrate temperatures of RT (a) and 450 °C (b).

For the topographic characterisation, atomic force microscopy (AFM) was performed at various regions on the CoFe_2_O_4_ thin films prepared at RT and 450 °C. Three-dimensional (3D) micrographs of representative AFM images of both samples are shown in Fig. 4. The AFM micrograph analysis reveals that the sample prepared at RT has higher roughness and porosity compared to films deposited at 450 °C. Thus, the sample prepared at RT has a higher surface-to-volume ratio, which provides an easy path for ion movement at the electrode–electrolyte interface, making it suitable for supercapacitor applications. The surfaces of samples prepared at RT and 450 °C have root mean square (RMS) roughness values of (20 ± 1) nm and (8.5 ± 0.5) nm, respectively.

### 3.4. Electrochemical studies

#### 3.4.1. Cyclic voltammetry study

**Fig. 5:**
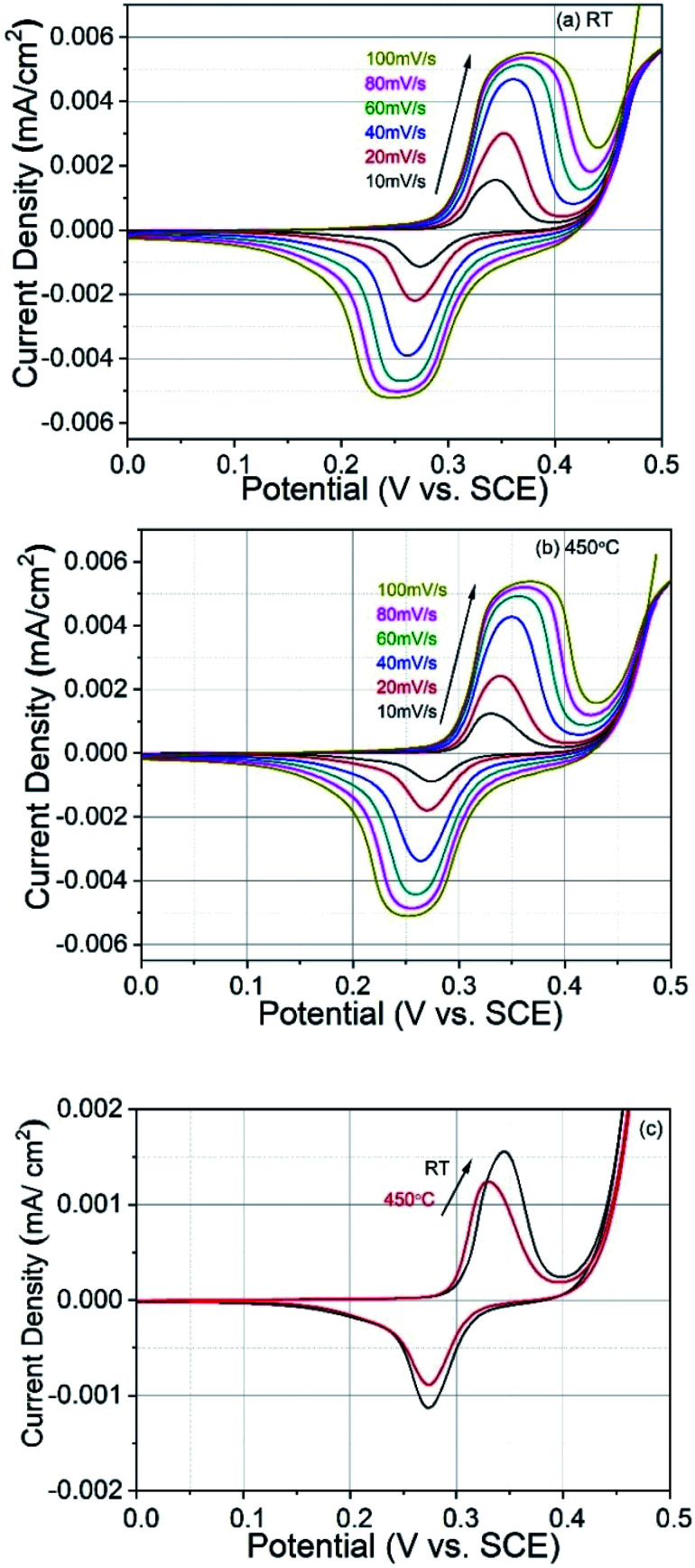
Cyclic voltammograms at different scan rates of CoFe_2_O_4_ electrodes prepared at (a) RT and (b) 450 °C. (c) CV plots of both electrodes at 10 mV s^−1^ scan rate for comparison.

To study the supercapacitive behaviour of the pulsed laser deposited CoFe_2_O_4_ thin films prepared at different substrate temperatures, cyclic voltammograms (CV) in the alkaline medium were measured using 1 M KOH solution as the electrolyte. The effect of the scan rate on specific capacitances was investigated and Fig. 5(a) and (b) show the variation of voltammetric current at different scan rates for films prepared at RT and 450 °C, respectively. The electrochemical measurements of both types of CoFe_2_O_4_ electrodes clearly show a highly pseudo-capacitive nature. The voltammetric currents are increase with the scan rate, and this behaviour is similar to that of an ideal capacitor.^39^ The CV curves correspond to the conversion between different iron and cobalt oxidation states. The redox reaction of CoFe_2_O_4_ was mainly ascribed to the redox pairs Co(iii)/Co(ii), Co(iv)/Co(iii), and Fe(vi)/Fe(iii).^40,41^ The superposition of the redox processes corresponding to the transitions of these redox pairs resulted in the broad CV peaks.^42^ The electrode materials with intercalation pseudocapacitance can demonstrate non-negligible CV peaks.^43,44^ CV curves similar to our results were recently published and interpreted in terms of supercapacitor behaviour.^19,28,45^ Both films studied here showed good adherence to the substrate and these electrodes also did not crack after electrochemical measurements. Fig. 5(c) shows CV recorded at 10 mV s^−1^ for both electrodes and it can be seen that the electrode prepared at RT shows larger charge storage capacity as compared to the electrode prepared at 450 °C substrate temperature. The observed better charge storage capacity of RT prepared electrode might be due to favoured ion intercalation/deintercalation related to the poor crystallinity. The crystalline quality of of the 450 °C electrode is better, as depicted in the XRD study, which restricts the ion intercalation/deintercalation.

#### 3.4.2. Galvanostatic charge–discharge study

**Fig. 6:**
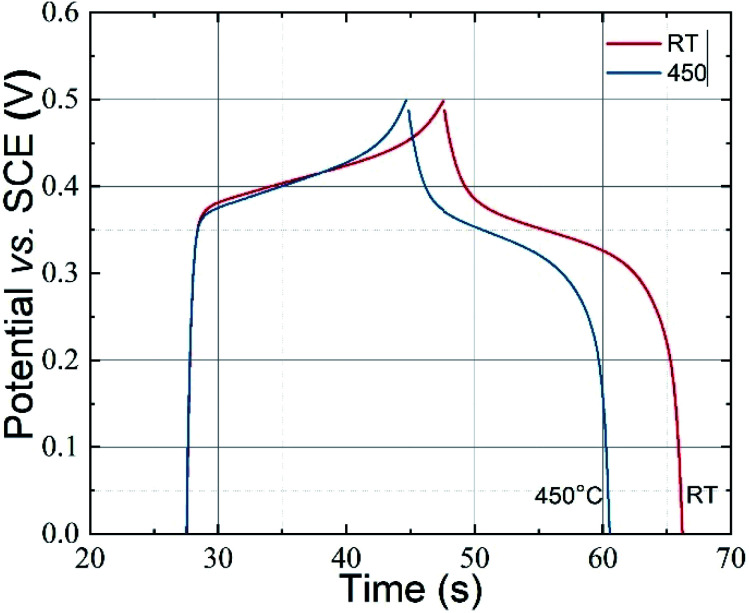
Galvanostatic charge–discharge curves for the CoFe_2_O_4_ electrodes prepared at RT and 450 °C in 1 M KOH electrolyte at 0.5 mA cm^−2^ current density.

To understand the capacitive nature of the CoFe_2_O_4_ electrodes, galvanostatic charge–discharge (GCD) measurements were carried out. Fig. 6 shows the charge–discharge curves of CoFe_2_O_4_ electrodes prepared at RT and 450 °C in a 1 M KOH solution at a current density of 0.5 mA cm^−2^. As can be seen in the figure, the discharge curves for both types of electrodes are nonlinear. Such behaviour was already reported for CoFe_2_O_4_ electrode materials used for supercapacitor applications.^28,45^ According to Elkholy *et al.*, such nonlinear discharge curves indicate that the capacitive performance is due to pseudocapacitance.^19^ This can be attributed to the electrochemical adsorption–desorption reaction at the electrode–electrolyte interface.^46^ The specific capacitance calculated according to eqn (1) from the GCD curves for CoFe_2_O_4_ thin films deposited at RT and 450 °C was about 777.4 F g^−1^ and 258.5 F g^−1^, respectively, at 0.5 mA cm^−2^ current density, where the mass loading of the CoFe_2_O_4_ electrodes deposited at RT and 450 °C is 0.0305 mg and 0.0419 mg, respectively. The higher capacitance of films prepared at RT might be due to the higher surface area of the rough electrode material. Higher surface area could facilitate the electron transfer between the electrolyte and electrode and a greater wettability of the electrode during the charge/discharge process. In addition, amorphous materials can provide an easier pathway for the intercalation and deintercalation of charges, which improves the charge-transfer.^47^ The energy density and power density of CoFe_2_O_4_ films prepared at room temperature were calculated by the equations^19^2
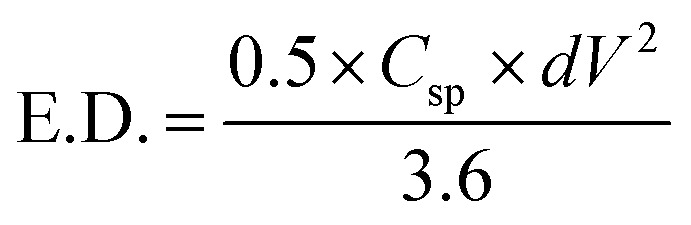
and3
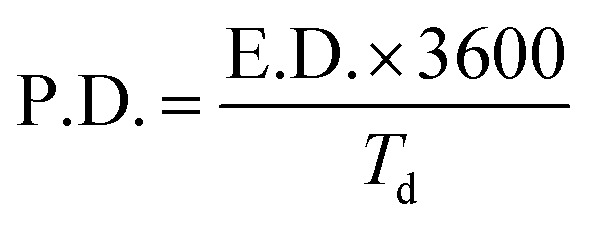
where E.D. is energy density, P.D. is power density, *C*_sp_ is specific capacitance, *dV* is a potential window of the discharge curve (here *dV* = 0.4 V), and *T*_d_ is the discharge time. The CoFe_2_O_4_ films deposited at room temperature exhibit an excellent power density (3277 W kg^−1^) and energy density (17 W h kg^−1^) compared to previously reported ferrite-based materials, as shown in Table 1. The values obtained for the power density lies in the supercapacitive regime in the Ragone plot shown in ref. 43. This further confirms the supercapacitive nature of the investigated electrodes.

**Table 1:** Comparison of supercapacitive performances of various ferrite electrodes

Material	*C*_sp_ (F g^−1^)	E.D. (W h kg^−1^)	P.D. (W kg^−1^)	Ref.
MnCoFe_2_O_4_	670	3.15	2250	19
CuCoFe_2_O_4_	397	3.53	198	48
ZnFe_2_O_4_	471	4.47	277	49
MnFe_2_O_4_	245	12.60	1207	50
CuFe_2_O_4_–graphene	577	15.80	1100	51
CoFe_2_O_4_	429	10.68	—	28
**CoFe**_**2**_ **O**_**4**_	**777**	**17.00**	**3277**	**Present work**

#### 3.4.3. Electrochemical impedance study

**Fig. 7:**
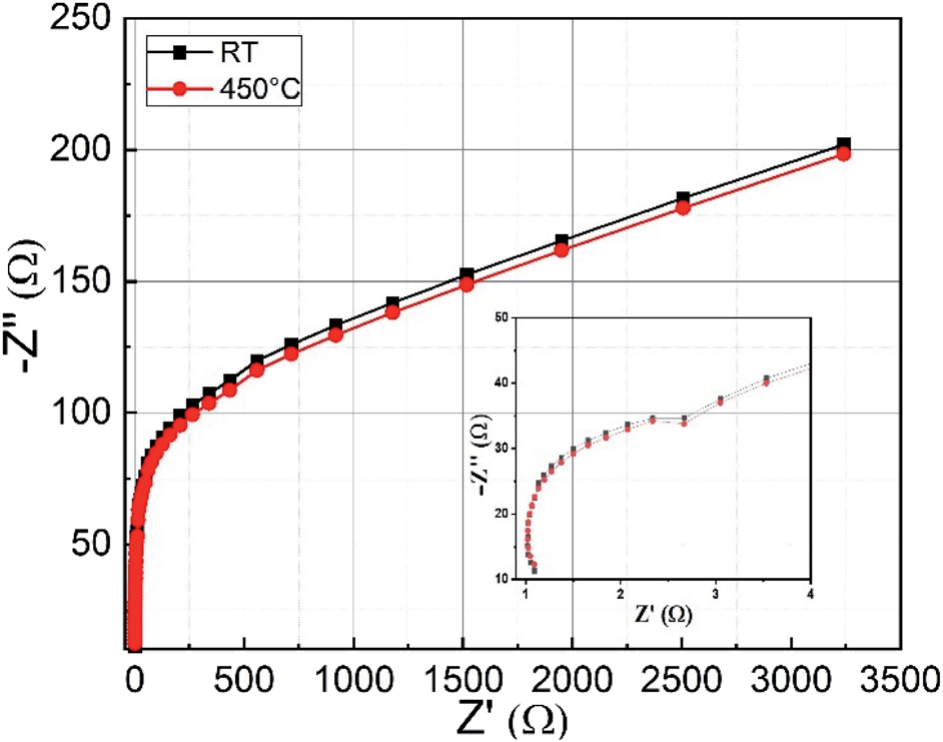
The Nyquist impedance plots for CoFe_2_O_4_ electrodes prepared at RT and 450 °C. The inset shows a magnified image of the higher frequency region.

Electrochemical impedance spectroscopy (EIS) is an excellent tool to get information about series resistance and charge transfer resistance of a material. Fig. 7 shows the Nyquist plots of CoFe_2_O_4_ thin films prepared at RT and 450 °C measured in 1 M KOH electrolyte. The spectrum is taken in the frequency range from 1 Hz to 10 kHz. In the high-frequency region, the intercept with the real part (*Z*′) represents the combined solution resistance (*R*_s_), which contains the ionic resistance of the electrolyte, the intrinsic resistance of active material, and the contact resistance.^28^ The semicircle shown in the inset represents the charge-transfer process at the electrode–electrolyte interface. Both types of samples show low series resistance (≈*R*_s_ = 1.1 Ω) and charge transfer resistance (≈*R*_ct_ = 1.5 Ω). In the low-frequency region, a straight-line curve close to 45° with respect to the *Z*′ axis for both types of CoFe_2_O_4_ electrodes is observed, which is recognized as the Warburg impedance related to the diffusion and transport of counterions between the electrolyte (KOH) and the surface of the electrode material for the duration of the redox reaction.^28,52^

#### 3.4.4. Cyclic stability

**Fig. 8:**
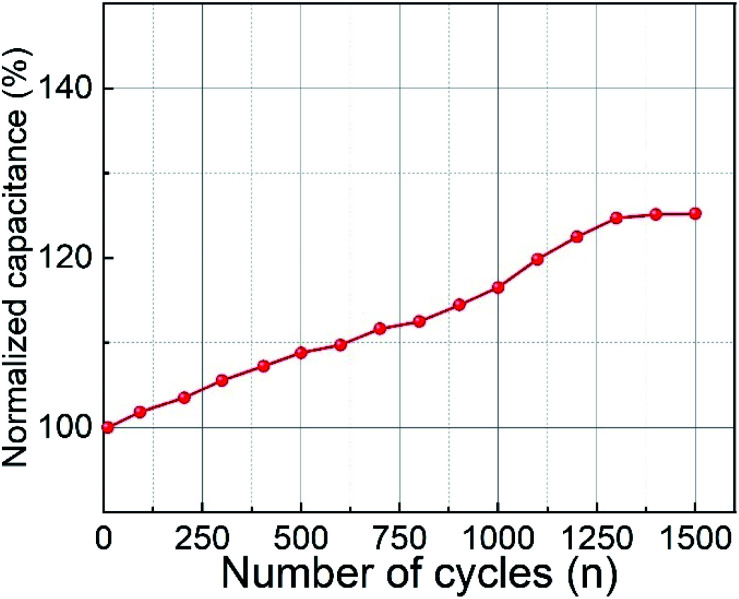
Cyclic stability of CoFe_2_O_4_ electrode prepared at RT at 0.5 mA cm^−2^ current density.

The cyclic performance of the CoFe_2_O_4_ electrode material prepared at RT was examined by GCD tests and is shown in Fig. 8. When the number of cycles was increased to 1500 cycles, a gradual increase in capacitance from 100% to 125% was observed. The increasing capacitance may be attributed to the activation of the electrode material with an increasing number of charge–discharge cycles.^53,54^ The activation of the electrode material enhances the participating electroactive surface area.^8,55^ Another possible explanation for the increasing capacitance might be structural changes within the CoFe_2_O_4_ films. Pseudocapacitive materials are known to undergo significant structural or microstructural changes during charge–discharge processes.^56^ A similar increase in percentage retention during measurement of cyclic stability was observed for zinc cobaltite by Mohamed *et al.*^57^ They obtained 119% retention capacity after 2000 cycles, while Aboutalebi *et al.* observed a 120.5% increase in capacitance after 1000 cycles for graphene oxide/multi-walled carbon nanotube composites.^58^

## 4. Conclusions

Cobalt ferrite thin films prepared by the pulsed laser deposition technique were used as electrodes for supercapacitor applications. The material formation was confirmed by XRD and Raman spectroscopy results. A specific capacitance of 777.4 F g^−1^ at 0.5 mA cm^−2^ current density was observed for the films prepared at RT, which is three times higher than that for the films prepared at 450 °C. This is attributed to the rough and porous surface of the films, which was confirmed by AFM. The films prepared at room temperature exhibited high power density (3277 W kg^−1^) and energy density (17 W h kg^−1^) values. The electrode material showed improved capacitance with increasing cycles due to the increase in the electroactive area. Keeping in mind the reproducibility and adherence of films prepared by PLD, the reported electrode materials could serve as a promising candidate for supercapacitor applications.

## Conflicts of interest

The authors declare that they have no conflict of interest.

## Supplementary Material
